# An Intergenerational Information and Communications Technology Learning Project to Improve Digital Skills: User Satisfaction Evaluation

**DOI:** 10.2196/13939

**Published:** 2019-08-09

**Authors:** Francesc López Seguí, Marc de San Pedro, Eva Aumatell Verges, Salvador Simó Algado, Francesc Garcia Cuyàs

**Affiliations:** 1 TIC Salut Social Generalitat de Catalunya Mataró Spain; 2 Centre for Research in Health and Economics Department of Experimental and Health Sciences Universitat Pompeu Fabra Barcelona Spain; 3 Universitat de Vic - Universitat Central de Catalunya Vic Spain; 4 Sant Joan de Déu Hospital Barcelona Spain

**Keywords:** active aging, digital inclusion, ICT program, intergenerational relationships, civic participation, community service

## Abstract

**Background:**

“Digital Partners” is an intergenerational information and communications technology learning project carried out in the municipalities of Vic and Centelles (Catalonia) from April to May 2018. Within the framework of the introduction of community service as a subject in secondary education, the Centre for Health and Social Studies (University of Vic) created a training space with 38 intergenerational partners (aged 14-15 years and >65 years), with the aim of improving the senior users’ digital skills in terms of use of smartphones and tablets, thus helping reduce the digital divide in the territory.

**Objective:**

The aim of this paper is to evaluate the satisfaction of both junior and senior participants toward the intervention and to explore its main drivers.

**Methods:**

Participants who volunteered to participate in the study were interviewed. Quantitative and qualitative data gathered in paper-based ad hoc surveys were used to assess participants’ satisfaction.

**Results:**

The experience shows a broad satisfaction of both junior and senior users. The project’s strengths include the format of working in couples; randomly pairing individuals by operating system; the ability to practice with the device itself; individuals’ free choice to decide what they wish to learn, develop, or practice; and the availability of voluntary practice material that facilitates communication and learning. With regard to aspects that could be improved, there is a need to review the timetabling flexibility of meetings to avoid hurrying the elderly and to extend the project’s duration, if necessary.

**Conclusions:**

This activity can serve to create mutual learning through the use of mobile devices and generate security and motivation on the part of the seniors, thus reducing the digital divide and improving social inclusion.

## Introduction

**Figure 1:**
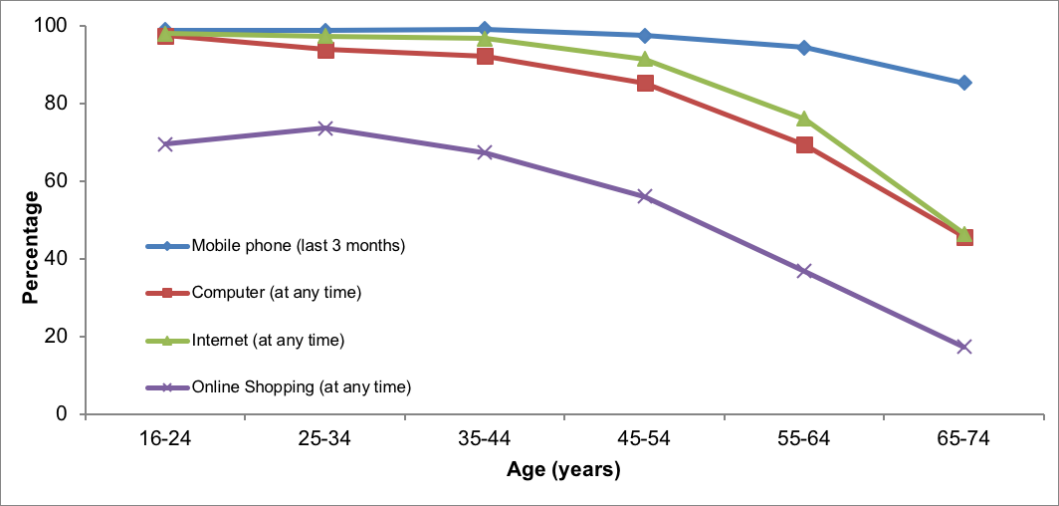
Use of different forms of information and communications technology according to age, as a percentage of the total population. Source: Survey on Equipment and Use of ICT technologies in Households [1].

The fourth industrial revolution is increasingly revealing its implications, potentially widening the digital divide and increasing inequality and social atomism. The evident emergence of the use of mobile devices has resulted in some surprising statistics: 99% of young people between the ages of 16 and 24 years in Spain have used a mobile phone in the last 3 months (Figure 1) [1]. More surprisingly, and unlike other technologies, the use of mobile devices is similar between generations: The uptake of mobile devices between older and younger generations only decreases by 15%, whereas other technologies have a significantly lower acceptance rate among people of older age, suggesting that the use of mobile technology by the elderly may not differ much from that of young people, and this trend is important in relation to forecasts of demographic ageing for developed countries [2,3].

The context of this study is Osona, a region located in the northeast corner of the Central Catalan Depression. With 150,000 residents, its population structure includes medium and small areas affected by social isolation and difficulties associated with an ageing population [4,5]. In this region, the 2017-2018 school year included community service as a voluntary academic activity in the secondary education centers [6]. The Centre for Health and Social Studies designed the project and invited two secondary education schools to participate together with the corresponding University Extension Classes (educational centers for the elderly).

Social common perceptions and assumptions about older people are mistakenly based on stereotypes [7,8]. According to the data presented, new technologies may be encouraging structural changes in the seniors’ social and relational behavior. Thus, it is necessary to understand and take into account their facilitating and acceptance factors. The literature shows that the adoption of mobile devices by older people responds to motivations that are very similar to those affecting young people and adults (Textbox 1) [9,10,11].

**Motivations and obstacles to the use of mobile devices by the elderly, based on the study by McGaughey [ 9].**

**Motivations:**

- Enjoyment/fun
- Expressivity
- Self-awareness
- Prevention (personal)
- Security (personal)
- Ability to communicate with others
- Freedom
- Autonomy
- Usefulness
- Social influence
- Availability of the service
- Value of the service
- Characteristics of the product
- Reduced costs

**Obstacles:**

- User interface
- Program interface
- Size of the device
- Shortcomings in ease of use
- Complexity of the device
- Availability of the service
- Costs (device and service)
- Availability of infrastructure
- Loss of privacy
- Physical abilities
- Cognitive abilities
- Lack of confidence
- Lack of training/knowledge

## Methods

The junior users were 42 young people between the ages of 14 and 15 years, mostly girls (65%), who were voluntarily recruited by their teachers. The schools had contacted the promoters because they were interested in a collaborative activity. These are centers that collaborate throughout the year with other intergenerational activities. The senior users were 38 individuals aged over 65 years, mostly women (75%), who were voluntarily recruited from the aforementioned University Extension Classes (February/April 2018). The juniors and seniors made up a total of 38 digital couples.

During the preparatory session, each junior received a document containing guidelines on content related to the use of mobile devices, divided into two blocks depending on the type of device (mobile phone or tablet) and three levels of difficulty (basic, medium, and advanced). A summary of the material is presented in Textbox 2.

**Structure and content of student training.**

**Theoretical module 1:**

Brief explanation of what aging is, what variables influence it, stereotypes, and opportunities for technological change.

**Exercise:**

Older people are shown in everyday situations with mobiles, and young people have to say what they see and what perceptions they have. It seeks to explore the potential and difficulties in the use of technology by senior citizens and break stereotypes. It encourages discussion about what we mean by seniors, where individual differences are given, and brainstorming about their motivations.

**Theoretical module 2:**

Aspects that should be taken into account to teach senior citizens how to use mobiles are listed below:

(1) the intergenerational relationship; (2) personal aspects (attitude, acceptance, empathy, assertive communication, values, and motivation); (3) the learning process (individual differences: age, physical and cognitive status, educational level, lived experiences, personal interests, knowledge, expectations, and needs); (4) technical aspects (content, simplify, facilitate help, encourage, and respect the privacy); and (5) practical guidelines.

**Exercise:**

Different objects from different generations are presented to introduce the subject of shared languages ​​and cultural meanings.

The material was specially prepared by the psychologist involved in the project, taking into account the following factors: the need to cover some of the most common uses of mobile devices, the degree of learning difficulty of each item, and the added value of the content in relation to the basic operation of the devices (for example, the optimization of battery usage). The material was provided as an optional ad hoc technical guide to be consulted in case the participants needed help in finding topics to work with in a particular order during the sessions. Content that might be sensitive or represent a degree of risk to the participants, such as actions requiring the use of a credit card, were excluded both from the material and the activity.

The sessions were based on the Intergenerational Mentor-Up philosophy and the Collaborative Learning methodology [12-17]. The advantages associated with this learning method at the academic, social, and psychological levels have been widely recognized, showing that greater performance in the use of mobile technologies favors social contact, reduces loneliness, and improves mental well-being [18-20]. The sessions were organized into two parts: in the first part, a psychologist conducted a training session for youngsters on active ageing, learning, and the pedagogy of digital skills among seniors. In addition, two specialists showed them case studies concerning the teaching and learning of digital competences.

The participants then conducted two or three “Digital Partners” sessions, lasting for about 1.5 hours, which were coordinated and supervised by the same psychologist. Students were asked to present themselves, ask how they could help, explore for how long the seniors had been using the device, how they had learned to use it, which functionalities they used the most, what they wanted to learn, and what their interests were in order to assess their needs and plan an answer; adjust the learning according to the person; show interest; and be friendly, empathetic, and respectful.

The pair worked with their own devices (it allows them to have hands-on experience and ask practical questions to resolve their real-life doubts) and were randomly paired on the first day. They remained the same throughout the sessions. Once participants formed by couples, if a special need was detected, couples were relocated according to the specifics on the basis of the advice of the student’s tutor (who knows the academic level, experience, and mastery of the language of young people).

During the recruitment process, users were asked which operating system (Android or IOS) they were most familiar with (in the case of juniors) or which system they used (in the case of the seniors) to ensure they possessed the necessary digital skills, so that the digital partners were the most suited according to their needs. The room in which the sessions were conducted was equipped with Wi-Fi and was set up especially for the activity, with the desks spread out as much as possible, each with two chairs, and labels announcing the type of software that was to be used. Each table was provided with support material for taking notes. In the absence of one of the seniors, the juniors were reassigned to an existing couple to provide extra help.

At the end of the last session, a voluntary, anonymous, ad hoc, nonvalidated, paper-based, 17-item questionnaire was filled out by all the participants who were able to freely devote as much time as they needed to answer the questions. This paper aims to outline the design and results of the project to understand the key determinants of success of the intervention in order to bring old and young people together in a shared learning activity. A descriptive analysis was carried out to distinguish between qualitative and quantitative data.

## Results

### Quantitative Evaluation

The variables and scores corresponding to the quantitative evaluation are shown in Table 1. The number of high scores is of particular interest (the lowest average score was 8.7/10), indicating both a possible high level of satisfaction with the intervention and a possible lack of critical consideration on behalf of the respondents, potentially because of peer influence when filling in the questionnaires.

**Table 1:** Average scores (out of 10) according to the question sets.

Set and question		Juniors	Seniors
**Teachers**			
	Teachers’ mastery of the subject	8.86	9.42
	Clarity and coherence in the presentation of the information	9.12	9.25
	Attention to personal enquiries and relationship with students	9.46	9.69
**Content**			
	The contents learnt are useful	9.03	9.45
	The materials used or recommended are useful for learning	9.48	9.09
	Suitability of the educational methodology, exercises and case studies	9.64	8.83
**Organization and facilities**			
	Information and attention received before the project	8.98	9.05
	Efficiency in resolving incidents, if any	9.58	9.3
	Operation of technical and audio-visual media	8.79	8.83
	Suitability of classrooms or laboratories (face-to-face sessions)	9.17	9.3
**Format and duration of the activity**			
	Duration of training (only applicable to the juniors)	8.71	N/A^a^
	Timetabling of sessions	9.2	9.58
	Duration of sessions	8.86	9.27
**Activity**			
	Belief that what you have learnt will be of personal benefit you	9.44	9.59
	The activity has met your expectations	9.38	9.46
Overall evaluation of the activity		9.48	9.55

^a^Not applicable.

### Qualitative Evaluation

The quantitative analysis consists of two forms of participation: in the first part, the participants are asked to identify the strengths and potential areas for improvement in the activity. These are summarized in Table 2.

The answers, which represent the main results of this experience, show that the activity had a positive impact on the juniors from the point of view of personal experience, interpersonal relations, and self-esteem; that the seniors considered the ability to use their own smartphone or tablet very useful and the involvement of only two people made it easy to deal with their questions and needs in a personalized way; and that both juniors and seniors value the fact that they always worked with the same partner. On the other hand, both groups of participants thought that the timing, duration, and scheduling of the sessions were aspects that could be improved.

**Table 2:** Summary of answers (verbatim) of the qualitative evaluation.

Open questions	Juniors	Seniors
What are the project’s advantages?	Meeting someone different Everyone benefits Learning about life Having the same partner That a child can help an adult	Being able to practice with your own mobile or tablet Having a private teacher Young people cheer you up Working with a partner and punctuality Having the same partner Being able to clear up doubts and needs Working with a partner
What would you change about the project?	Earlier Longer	Doing a course that lasts a whole term Having more sessions, since some topics are difficult More days (1 hour a day) Cover more in two days Poor acoustics in the classroom That we are given suggestions as to what to learn

**Summary of answers (verbatim) to the question, “Out of everything you’ve learnt, what 3 did you like the most?”**

**WhatsApp:**

- Sending WhatsApps
- Sending location
- Sending voice messages
- Taking photos
- Sending photos
- Creating groups
- Sharing YouTube videos
- Accessing archived messages

**Using a mobile phone:**

- Connecting to a Wi-Fi network Changing screen settings
- Creating widgets
- Using the camera
- Creating photo albums
- Connecting the tablet to the mobile
- Learning about the battery
- Setting alarms

**Other:**

- Sending emails
- Using YouTube
- Understanding and learning how to use Instagram
- Learning how to share content between different platforms
- Understanding notifications

In the second part of the qualitative approach, a random selection of participants were asked a question while they were participating in the sessions: The senior users (25 respondents) were asked, “Out of everything you’ve learnt, which 3 items did you like the most?” The answers are summarized in Textbox 3, highlighting the learning elements related to the use of WhatsApp and using the phone, in general, as well as a general feeling of having gained confidence in using the device.

The junior participants were also individually chosen at random to answer the following question: “What have you got out of this activity?” We identified three areas of consensus: first, working on stereotypes (with comments such as “some of them don’t have children or grandchildren,” “some of them are fun,” or “some of them know a lot about using a mobile phone”); second, the sense of personal fulfilment (for example, “it makes you feel good to help,” “happiness,” “it's nice to help,” “really good”); and finally, it shows the importance of the fact that the activity allowed them to take decisions in guiding their partner's learning, face up to challenges, and learn new things (eg, “they’ve asked me something I don’t know, so I’ll look into it for the next session”). The majority stated that they made use of the user’s guide of suggested topics and that they referred to the contents at a basic level. Thus, on the one hand, the seniors who signed up had a very basic knowledge regarding the use of mobile devices and, on the other hand, the uses and contents that interested them the most were those that allowed them to have better control over the device.

In keeping with the quantitative evaluation, the results generally show a very high level of satisfaction and highlight its main drivers. From the point of view of personal experience, the participants emphasized the significance of learning and collaborating with another individual. The activity’s strengths were the format of working in pairs; pairing people by operating system; the use of the participants’ own mobile phones; the freedom to decide what they wished to learn, develop, or practice; and the availability of voluntary practice material, which facilitates communication and learning. It has been shown that the activity can serve to create mutual learning through the use of mobile devices and generate security and motivation on the part of the seniors, thus reducing the digital divide and improving social inclusion [21-24].

## Discussion

### Principal Findings

The quantitative results presented can be taken into account in terms of *positively* assessing to what extent the objectives have been met, but they unfortunately do not provide useful insights on the success drivers of the interventions. Nuances and critical aspects to be taken into account in further studies can be derived only from the qualitative evaluation, which is in line with previous literature showing that seniors can benefit from the learning process and improve their digital self-efficacy and that the intervention can be successful in building an intergenerational bridge [25-27]. Previous analysis also shows that those benefits are not systematic and depend on the meaningfulness of the activities, organization of the program, and participants’ knowledge of the other generation [28]. This can explain the assessed success of our program, which emphasized the student training and material preparation.

### Limitations

We were unable to analyze variations in the effectiveness of our intervention due to resource limitation. Therefore, further studies attempting to replicate the intervention described here should analyze these variations, depending on the previous senior digital literacy and age. Moreover, constant developments in mobile devices (versions, design, capacities, etc) suggest that such training, as that described herein, should have a longer continuity to better assess the effect of the intervention over time. In addition, the questionnaire should be further adapted to capture all the critical analyses of the youngsters.

### Implications and Recommendations

The activity’s success is based on its design, which takes into account the needs and abilities of the participants: location, facilities and design of the spaces, training young people receive, and materials used in conducting the sessions, paying special attention to the seniors’ learning environment so that they can take full advantage of opportunities to improve their knowledge and skills, and the learning environment of the young people so that they can carry out their tasks effectively while also enjoying learning. Similarly, attention must be paid to certain requirements that may affect development of the activity: connectivity, noise level in the classroom, dividing the participants into pairs, the duration and timetabling, and the type of training (which should be personalized and adapted to the users’ needs, although they should also have material available to them as a guide if they so wish).

Many functions of so-called smartphones have been designed without taking into account the needs of senior users; nevertheless, seniors are interested in improving their knowledge and learning to use technology to take advantage of what it offers, as shown by their motivation in carrying out the activity, but also in relation to the dynamics of the session (personalization) and the learning contents (basic). This project addresses the risk of a digital divide, which can increase social inequality. It does so by empowering and transmitting skills. The intergenerational approach leads to mutual and fundamentally bidirectional learning and allows older people to resume their traditional role as transmitters of knowledge.

## Abbreviations

N/A: not applicable
